# CPAC: Energy-Efficient Data Collection through Adaptive Selection of Compression Algorithms for Sensor Networks

**DOI:** 10.3390/s140406419

**Published:** 2014-04-09

**Authors:** HyungJune Lee, HyunSeok Kim, Ik Joon Chang

**Affiliations:** 1 Department of Computer Science and Engineering, Ewha Womans University, 52 Ewhayeodae-gil, Seodaemun-gu, Seoul 120-750, Korea; 2 Department of Electronics and Radio Engineering, Kyung Hee University, 1732 Deogyeong-daero, Giheung-gu, Yongin-si, Gyeonggi-do 446-701, Korea; E-Mails: qrivetree@khu.ac.kr (H.S.K.); ichang@khu.ac.kr (I.J.C.)

**Keywords:** selective compression, data collection, energy efficiency, sensor networks

## Abstract

We propose a technique to optimize the energy efficiency of data collection in sensor networks by exploiting a selective data compression. To achieve such an aim, we need to make optimal decisions regarding two aspects: (1) which sensor nodes should execute compression; and (2) which compression algorithm should be used by the selected sensor nodes. We formulate this problem into binary integer programs, which provide an energy-optimal solution under the given latency constraint. Our simulation results show that the optimization algorithm significantly reduces the overall network-wide energy consumption for data collection. In the environment having a stationary sink from stationary sensor nodes, the optimized data collection shows 47% energy savings compared to the state-of-the-art collection protocol (CTP). More importantly, we demonstrate that our optimized data collection provides the best performance in an intermittent network under high interference. In such networks, we found that the selective compression for frequent packet retransmissions saves up to 55% energy compared to the best known protocol.

## 1. Introduction

According to a report by U.S. Department of Energy (DOE) and the U.S. Environmental Protection Agency (EPA), commercially available technologies can help to achieve energy savings up to 25% [1]. Wireless sensor networks (WSNs) have been regarded as one of the most promising solutions to reduce energy consumption by monitoring environment (e.g., energy usage over time) and dynamically adjusting network operation with respect to energy efficiency. Accordingly, an energy-aware network design has been a key topic in both sensor network research and industry applications.

There have been various approaches in the physical layer [2–4], Media Access Control (MAC) layer [5–9] and network layer [10–15] to reduce the energy consumption of sensor network applications. We consider that data compression may provide another possibility to further improve energy savings on top of the existing techniques in sensor networks. Previous work shows that in WSN, radio transmission energy occupies a substantial portion of the total energy consumption of a sensor node [16]. It is obvious that data compression can contribute to reducing radio transmission energy, prolonging the battery life time of the WSN. However, most data compression algorithms require a large memory size for computation, limiting the application to a WSN. The authors of [17] handle this challenge by presenting a computationally-efficient compression algorithm, named Sensor Lempel-Ziv-Welch coding (S-LZW), which fits into some limited RAM in real sensor motes. Furthermore, researchers developed another compression algorithm for a WSN, namely Run-Length Encoding with Structured Transpose (RLE-ST) [17]. Compared to S-LZW, this algorithm takes less computation time, but instead, leads to a lower compression ratio.

In this paper, we study the problem of minimizing network-wide energy consumption for data collection from data sources at a data sink in sensor networks. Given some information of the temporal and spatial correlation of sensor data, the delay sensitivity of data and previous network routing patterns to the sink, we aim to obtain an energy-optimal joint compression and routing mechanism. We assume that data sources and sensor nodes are stationary. Furthermore, it is assumed that each datum from a data source has some tolerated latency, meaning that as long as data are delivered to the sink within the given latency constraint, the network application does not experience any service interruption.

We solve the problem of data collection at a sink by selectively choosing a subset of nodes that should perform compression and corresponding compression algorithms under the given latency constraint. We employ data compression algorithms into designing an energy-efficient data collection protocol. We note that data compression incurs some computation delay; energy savings, on the other hand, can be obtained. As discussed earlier, S-LZW provides more energy savings compared to RLE-ST at the expense of more delay. Hence, depending on a given latency requirement, a suitable compression algorithm can be applied to the underlying routing mechanism on the way to the sink. In this work, we assume the scenario where only two compression algorithms are available, which are S-LZW and RLE-ST. This is due to the fact these are the most well-known compression algorithms for WSN. However, our proposed algorithm can be easily applicable to other scenarios with different compression algorithms, too.

As discussed earlier, S-LZW provides more energy savings compared to RLE-ST at the expense of more delay. We explicitly consider this trade-off relation between latency and energy efficiency and formulate it into a compress decision optimization model of binary integer programs. We develop this optimization to take into account sensor data correlation, latency requirements and route information. The binary integer programming aims to find a joint optimal decision regarding the following things (under latency constraints): (1) which sensor nodes should execute compression; and (2) which compression algorithm the selected sensor nodes should use, to minimize energy consumption across the network.

First, to quantify the trade-off relation between latency and energy savings for each respective compression algorithm, we make extensive measurements on how the data length for transmission (TX) and reception (RX) affects radio energy, computation energy and delay. Based on these, we construct an empirical model of radio energy savings (as a benefit of data compression) and of the resulting computation energy cost and processing delay (as corresponding penalties).

Second, given the derived empirical model and a latency constraint, we design an energy-efficient collection protocol that utilizes the aforementioned optimization model. For the underlying collection tree-based routing, we take the state-of-the-art collection protocol (CTP) [18]. The proposed energy-optimized collection protocol consists of two phases: (1) the learning phase to learn dynamic route path information from each data source and data compressibility from the collected data and extracting necessary parameters for optimization; and (2) the optimized collection phase to apply the computed compression decision at each sensor node obtained from the optimization and allowing compressed data to be delivered to the sink.

We evaluate our optimized data collection protocol with adaptive compression (CPAC) in relation to the unmodified CTP [18] on two different real-world sensor datasets, Intel Lab data [19] and SensorScope [20]. Simulation results demonstrate that the addition of our optimization algorithm to CTP improves the original CTP with an energy savings of 47%. More importantly, in an intermittent network under high interference, where considerable retransmissions occur, an energy savings of up to 55% is achieved.

The rest of this paper is organized as follows: After discussing related work in Section 2, we present the system model in Section 3. In Section 4, we show our model of the energy and delay costs of compression algorithms, and Section 5 proposes our optimization algorithm. After we present the evaluation results of our approach in Section 6, we conclude this paper in Section 7.

## 2. Related Work

There have been energy savings efforts through data compression in a WSN. We classify related works into the following two categories: (1) the redesign of compression algorithms for sensor motes with low-end CPU and limited RAM space [17,21,22]; and (2) the optimization of network routing with compression algorithms [23,24].

### 2.1. Compression Algorithm Design

Researchers in [22] propose a power-aware coding scheme that exploits the spatio-temporal correlation of sensor data for energy savings. While taking the existing source coding schemes, Set Partitioning In Hierarchical Trees (SPIHT) and Extended Set Partitioning In Hierarchical Trees (ESPIHT), to reduce redundancy on the spatio-temporally correlated data, the authors optimize the source coding scheme with channel coding based on Automatic Repeat reQuest (ARQ) with dynamic power allocation. The proposed scheme leads to more than 60% energy savings. A lightweight compression algorithm, S-LZW, is introduced in [17]. This work tailored the original LZW algorithm for small memory usage in a WSN by finding fitting compression parameters, such as dictionary size and data size. While the authors also implement a simple RLE-ST compression algorithm for a WSN as a counterpart to S-LZW, they suggest an instructive algorithm selection guideline depending on the radio hardware, network size and the structure of sensor data. Marcelloni and Vecchio [21] present an entropy-based compression algorithm that uses the baseline JPEG algorithm for compressing DC (direct current) coefficients. Evaluation results show that the proposed algorithm outperforms S-LZW in terms of the compression ratio and computational complexity.

Our work does not propose a new compression algorithm for a WSN; rather, we employ existing compression algorithms, such as S-LZW and RLE-ST, for optimizing the usage of compression at a higher network level through a network-wide compression decision.

### 2.2. Routing Optimized with Data Compression

Several previous works proposed network-level routing schemes supplemented with compression algorithms. Pipelined In-Network COmpression scheme (PINCO) [23] is an in-network compression scheme that reduces redundant data transmissions. Using some tradeoff of the delay, an intermediate aggregation node collects data from leaf nodes at the buffer. After a while, it classifies common parts from the buffered data and transmits the common data parts only once, enabling a more energy-efficient data collection. Some researchers propose the data funneling scheme [24], which achieves energy-efficient packet transmission through combining two contributing components: (1) packet aggregation; and (2) data compression. By allowing a group of sensors to send only one data stream to a data sink, the proposed packet aggregation scheme reduces the probability of packet collisions and improves energy savings. Further, the coding-by-ordering compression technique is combined; this suppresses some of the packets, while indicating the dropped information instead with encoded values from the ordering of the remaining packets.

Our work more closely falls into this category. Even though our work also proposes a network-level optimization using data compression techniques, we provide clear answers for the following more fundamental questions: how and where should the data compression technique be used for energy-efficient network routing, while satisfying the given latency constraint?

## 3. Problem Formulation

This work considers the problem of energy-efficient data collection in sensor networks. The main objective of this paper is to minimize radio and computation energy consumption, while meeting the given latency constraint. We consider various factors of radio and computation energy consumption dynamics before and after applying compression, the hop distance to the data sink and a given latency deadline. Obviously, data compression reduces the number of bytes to transmit and, hence, the transmission energies of each sensor node. However, this also results in considerable penalties with respect to computation energy and network latency. The S-LZW and RLE-ST algorithms show fully different characteristics regarding the above trade-off relation (shown in Section 4).

We consider a network application of timely delivery to the data sink while reducing the overall energy consumption throughout the network and fully exploiting the allowed packet delivery time. The following scenario summarizes how the network can exploit the above trade-off relation for an energy savings benefit. If data require a very tight end-to-end latency from source to sink, the source itself and the relay nodes would not, rather, take compression to meet the tight deadline, although the intact data transmission incurs a large amount of radio energy cost. On the other hand, if data have a somewhat tolerated delay, there is a possibility to optimize the usage of data compression network-wide; some intermediate relay nodes with many leaf nodes in a network tree could be selected for compression, as long as the incurred computation delay does not affect the promised end-to-end latency requirement much. Furthermore, if there are other data compression candidates to choose from, the network needs to be optimized more seriously by considering different compression cost in delay, computational energy, compressibility and the corresponding radio energy consumption.

**Figure 1.**
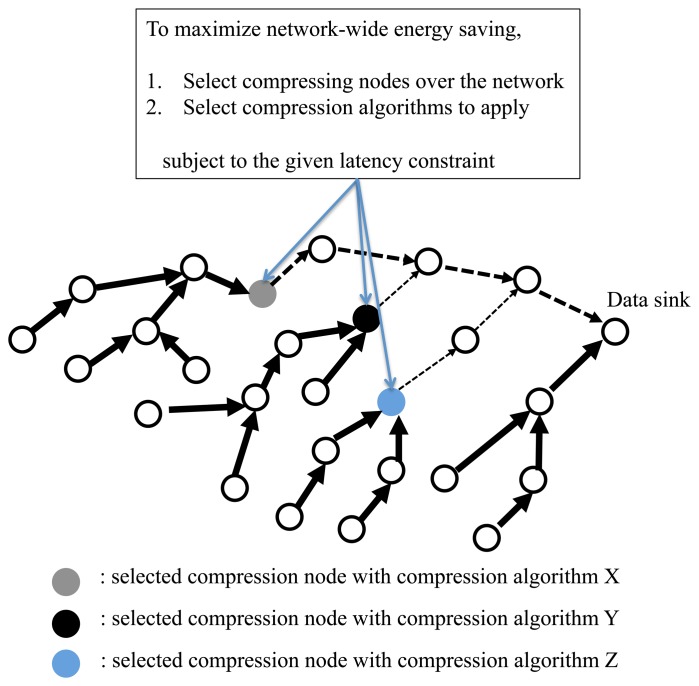
The overview of sensor data collection based on selective data compression optimization. Compressing nodes (three nodes selected) and their algorithms to be applied (among various choices) are selected to maximize network-wide energy savings under the given latency constraint.

We assume that in a normal data collection mode (where no data compression is applied), a data sink receives routing path information from data sources. We also consider route variations resulting from link dynamics. The problem of energy-efficient data collection with data compression techniques can then be described as finding a network-wide optimal decision of which relay sensor nodes are allowed to compress data using which compression algorithm with respect to a given packet deadline requirement, as illustrated in Figure 1. After informing about the decision information of the network, the data collection protocol switches to the optimized data collection mode that uses selective data compression, leading to significant energy savings throughout the network.

## 4. Modeling Energy and Delay Costs of Compression Algorithms

In this section, we construct empirical models of radio energy savings for existing compression algorithms and of the resulting computation energy cost and processing delay. We take existing compression algorithms designed for sensor networks, *i.e.*, S-LZW and RLE-ST [17]. We compare radio energy consumption before and after each compression and decompression with respect to data size. We also measure corresponding penalties: incurred computation energy and processing delay upon applying each different compression algorithm. The derived models are employed as inputs for designing an energy-optimized collection scheme in Section 5.

### 4.1. Experiment Environment

We describe the dataset, compression algorithms and platforms used in our experiments regarding data compression.

#### 4.1.1. Dataset

We use real sensor dataset: (1) Intel Lab data [19]; and (2) SensorScope [20]. Intel Lab data consists of a time stamp, humidity, temperature, light and voltage values from 54 sensors deployed in the Intel Berkeley Research Lab. SensorScope data comprises timestamps and the solar radiation environment information from an outdoor deployment.

#### 4.1.2. Compression Algorithm

We use compression algorithms, which are: (1) S-LZW; and (2) RLE-ST [17]. S-LZW is one of the most intensive compression algorithms well-fitted for a WSN. At the other extreme of compression algorithm is the RLE-ST algorithm, as a counterpart to S-LZW, because it is a very simple algorithm that works well for many consecutive data elements.

#### 4.1.3. Measurement Platform

We measure radio energy consumption for TX and RX based on PowerTOSSIM [25] for TinyOS 2.x. The computation energy consumption for compression and decompression is measured based on the running time of each algorithm targeted at ATmega128L, which is a micro controller unit (MCU) used in many mote-class sensor nodes.

### 4.2. Performance Analysis

We compare total energy consumptions comprising radio energy and CPU energy before and after applying compression with S-LZW and RLE-ST with respect to data size. We also compare corresponding processing delays, which eventually affect the end-to-end latency.

#### 4.2.1. Per-Hop Energy Consumption

**Figure 2.**
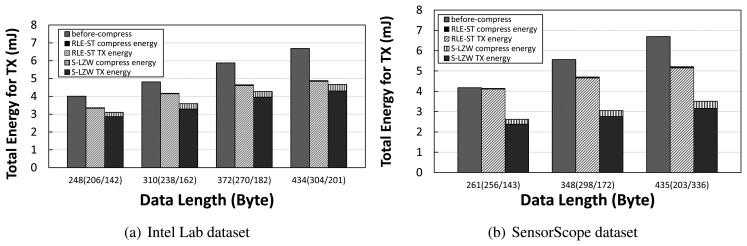
Per-hop transmission energy cost measurement in millijoules before/after compression with S-LZW and RLE-ST.

**Figure 3.**
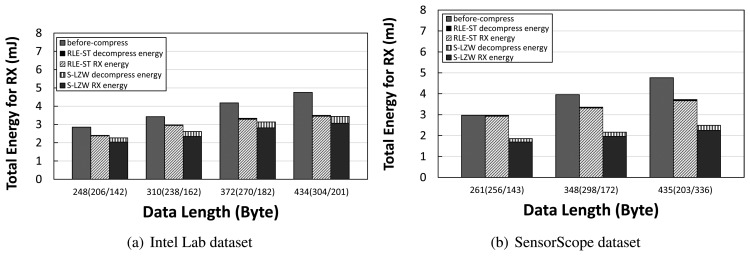
Per-hop reception energy cost measurement in millijoules before/after compression with S-LZW and RLE-ST.

We measure energy consumption before and after compression in a simple topology consisting of sender and receiver. In a normal packet transmission scenario, *i.e.*, before-compression, radio energy is consumed for transmitting the uncompressed data. On the other hand, if we apply data compression, *i.e.*, after-compression, a sender transmits a packet with smaller data length, reducing the radio energy. Since the packet length in TinyOS is limited to 114 bytes, packets having larger than such a limitation are split into multiple fragments. We consider this problem for our measurements. We also measure the computational energy for running the data compression algorithm. We show total energy consumption for the TX case in Figure 2 and for the RX case in Figure 3. For both datasets, it is shown that the total energy consumption for transmission and reception with after-compression is less than that of the normal packet transmission and reception, *i.e.*, before-compression. The amount of reduced radio energy for transmitting and receiving packets with the decreased data length due to compression is larger than the amount of increased computation energy for compression in TX and for decompression in RX. A more intensive compression algorithm, S-LZW, leads to more energy savings in total compared to RLE-ST, although the computation energy of the S-LZW algorithm is larger than that of the RLE-ST algorithm. These quantitative results demonstrate that there is a practical need of data compression that benefits from greatly reducing the energy consumption in sensor networks.

#### 4.2.2. Computation Delay

**Figure 4.**
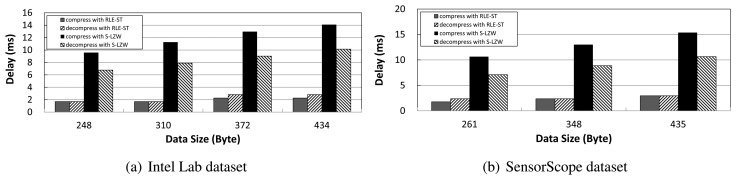
Compression and decompression delay in milliseconds with respect to the data size for S-LZW and RLE-ST.

We measure the computation delay of compression and decompression with S-LZW and RLE-ST. As shown in Figure 4, compression and decompression algorithms come with some overhead of processing delays. S-LZW has more processing delay compared to RLE-ST. This is due to the fact that a more intensive compression tends to result in a larger number of computations. When compressing or decompressing with S-LZW, it takes at least around 10 ms for their computation. This implies that in the case of packet delivery with a tight latency limit, taking S-LZW may cause failures of on-time packet delivery.

In summary, we show a trade-off between energy savings and delay due to compression and decompression with quantitative analysis. The energy savings from the S-LZW algorithm is larger compared to RLE-ST, but S-LZW is penalized with a longer delay. We construct energy and delay models of per-hop energy consumption for before-compression and after-compression with S-LZW and RLE-ST, respectively. We use those per-hop performance results of before/after each compression algorithm as parts of the input data to optimize data collection in Section 5.

## 5. Collection with Adaptive Compression

In the data collection WSN application, various environmental data, such as temperature, humidity, seismic vibration, air pollution and structural health, are periodically or non-periodically collected. One of the most common ways to build such sensor network applications is to deliver collected data from sensor nodes to a data sink. The data sink pre-processes and extracts contextual information from raw data across the network.

Collected sensor data often include some redundant information over spatial-temporal space. Previous works [26–28] propose summary-based data collection, such that intermediate relay sensors make summary information by taking the average or so, and delivering it to the next hop node toward a data sink.

Our work aims to mitigate redundant data delivery for more energy-efficient data collection using data compression. Data compression reduces the data size to transmit and, hence, significantly lowers radio energy consumption for packet transmission and reception [17]. Radio energy consumption is known to be the most dominant part of energy consumption in sensor networks. However, there is a trade-off between energy and time: whereas data compression can reduce total energy consumption, it may incur an additional processing delay. If this property is leveraged in a smart way, the network can obtain remarkable energy savings, while satisfying other constraints.

In this paper, we focus on designing an energy-optimized data collection scheme by taking a selective data compression strategy in stationary sensor networks that can lead to a significant reduction in network-wide radio and CPU energy consumption. We formulate the problem of the joint compression node and type selection as a binary integer program. The proposed scheme finds the compression node and type that minimize the estimated total energy consumption for data collection at a data sink node.

We describe the overall network protocol in Section 5.1 and our proposed network optimization algorithm for greatly improving the energy-efficiency of data collection in Section 5.2.

### 5.1. Protocol

The proposed protocol of energy-efficient data collection consists of two phases: (1) the learning phase; and (2) the optimization phase. In the learning phase, a data sink receives route path information with dynamic link variations (from continuous collection) and also tries to calculate the compressibility for the received data. This is due to the fact that sensor data is dependent on the environment and sensor type. Then, the network optimization algorithm at the data sink (or a cluster connected to the data sink) uses the route and data compressibility information to calculate compression node and type. The sink node informs about the calculated compression decision of the network using a dissemination protocol. In the optimization phase, sensor nodes execute their activity based on such a decision and then relay this to their next hops.

We describe the proposed protocol using the following five steps:

1. **Normal data collection**. A data source sends the generated or collected data where the required QoS latency requirement, *T_max_*, for the data is embedded in the header. A data sink receives data from other sensor nodes across the network as usual. Any intermediate node, as well as a data source append their own ID information on top of the data over multi-hop paths, so that the data sink can be informed of the path taken by each packet, hence the path from the source to the sink.
2. **Compression node and type selection**. When the data sink receives raw data from each data source, the sink calculates the average compressibility for each applicable compression algorithm type. Using received route path information, the QoS latency requirement and the energy and delay models (from Section 4), a computer cluster connected to the sink node decides the compressing nodes and their compression algorithm types that minimize the estimated overall energy consumption. In case proper compression nodes and their compression algorithms cannot be found, we continue the normal data collection and decide whether to switch to the optimization phase later on. The optimization procedure is described in Section 5.2.
3. **Decision information dissemination**. Compression strategy, which is calculated in Step 2, is put in a table and disseminated to the network by an efficient dissemination protocol (e.g., the Trickle algorithm [29]). This table comprises intermediate relay node IDs and types to compress data from each data source ID.

**Algorithm 1:** Data relay procedure with selective data compression in optimized data collection mode.
**Data:** 1) Data on reception or generated at source, 2) Computed compression strategy table of (source node ID, compressing node ID, compress type)**Result:** Compressed data with the header updated or the same data from input, and then relaying the data// for retrieving src ID for data;srcID = data→header→srcID;// for retrieving compressed bit field for data;compressedBitField = data→header→compressedBitField;// for retrieving compression type for data;compressType = data→header→compressType;**if**
*compressedBitField* == *1*
**then**   // if data have already been compressed Relay the received data as it is;**end****else**   // if data have not been compressed **if** (*srcID, myID*) *is in the table while returning back with type t***then**     **Compress** the data with compression type *t*;     data→header→compressedBitField = 1;     data→header→compressType = *t*;     Relay the compressed data; **end** **else**     Relay the received data as it is; **end****end**
4. **Data relay with selective data compression**. As soon as a data relay receives the compression strategy table, the data collection mode transitions from normal collection to optimized collection. When the relay node receives data, this node checks the header to see if the data are compressed or not. If the data are not compressed, the node checks the compression node list of the compression table. If the header shows that the data are already compressed or the node is not in the compression list for the data source, the node just relays the data to the next-hop node. Otherwise, the node refers to the corresponding compression type and accordingly executes data compression. After finishing the data compression, the node sends the compressed data. Refer to Algorithm 1 for a more detailed procedure.
5. **Decompression at the data sink**. Through several relay nodes, the compressed data reach the data sink. If the data sink receives this data, this checks the compression type field written in the header. This field informs the sink of which compression algorithm was used for the received data. Using the informed algorithm, the sink decompresses the data. Refer to Algorithm 2 for the details.

**Algorithm 2:** Data reception procedure at the sink node with selective data decompression in optimized data collection mode.
**Data:** Received data at sink node**Result:** Restored original data from data source// for retrieving src ID for data;srcID = data→header→srcID;// for retrieving compressed bit field for data;compressedBitField = data→header→compressedBitField;// for retrieving compression type for data;compressType = data→header→compressType;**if**
*compressedBitField* == *1*
**then**   // if data have been compressed **Decompress** the received data with *compressType*; Reception of data from *srcID* is done;**end****else**   // if data have not been compressed Reception of data from *srcID* is done;**end**

### 5.2. Compress Decision Optimization

Our work does not propose a new compression algorithm; instead, between two existing compression algorithms for sensor networks, S-LZW and RLE-ST, we dynamically choose one suitable for each sensor relay with respect to the network QoS requirement.

Before a data source or an intermediate relay node sends a data packet, the data compression of the packet reduces the data size, thereby improving transmission. This also reduces the receiving energy for the next node. As a side effect, however, the data compression algorithm incurs an additional processing delay, which affects QoS latency, and computation energy cost.

It should be noted that an intensive data compression algorithm with a higher compression ratio results in larger radio energy reduction as a benefit and also incurs larger overhead in the delay and computation cost as penalties. On the other hand, a modest compression algorithm leads to a smaller benefit and a smaller penalty.

Considering the above aspects, we formulate the problem of selective compression decision across the network into a global optimization problem with binary integer programming. The proposed algorithm finds the optimal compressing node and type for data collection from each data source to a fixed data sink. The data sink (or a computer cluster connected to it) makes an optimization regarding the above problem, while satisfying QoS latency requirement *T_max_*.

The data sink is given energy and delay models of per-hop network performance with any applicable compression algorithm and route path information from each data source to the sink as input (according to the procedure in Section 5.1). The output of the optimization algorithm is a set of compression decision (source node ID, compressing node ID, compression type) as follows: *D* = {(*srcID*_1_,* compID*_1_, *compType*_1_), (*srcID*_2_, *compID*_2_, *compType*_2_), …, (*srcID_n_*, *compID_n_*, *compType_n_*)} where *compType_i_* ∈ {*Algorithm*_1_, *Algorithm*_2_, …, *Algorithm_m_*} assuming that *m* data compression algorithms can be applied.

To set up the optimization problem with binary integer programming, we introduce an indicator function, *I_src_i_,comp_k_,type_t__*, denoting whether the compressing node ID, *k*, is selected to compress with compression algorithm type *t* upon receiving data from data source ID *i* as follows:

$$I_{src_{i},\text{com}p_{k},\text{typ}e_{t}}=\{\begin{array}{l} \begin{array}{ll} 1 & \text{if selected},\\ 0 & \text{otherwise}. \end{array} \end{array}$$

We define another indicator function to specify source nodes that do not execute any compression, *J_src_i__*:

$$J_{src_{i}}=\{\begin{array}{ll} 1 & \text{in case of no compression for srcID}\;i,\\ 0 & \text{otherwise}. \end{array}$$

Note that *I_src_i_,comp_k_,type_t__* for all *k*, *t* is mutually exclusive with *J_src_i__* (*i.e.*, Σ*_k,t_ I_src_i_,comp_k_,type_t__*+*J_srci_* = 1).

Based on these indicator functions, we define the objective function to minimize the network-wide energy consumption for radio and CPU computation and formulate the problem as a binary integer program as follows:

(1) $$\begin{array}{cc} \text{minimize} & \left(\underset{k,t}{\text{∑}}{\bar{E}}_{T}\cdot h_{i\to k}+E_{C,t}+E_{T,t}\cdot h_{k\to \mathrm{dst}}+E_{D,t}\right)\cdot \\ & I_{src_{i},\mathrm{com}p_{k},\mathrm{typ}e_{t}}+\left({\bar{E}}_{T}\cdot h_{i\to \mathrm{dst}}\right)\cdot J_{src_{i}} \end{array}$$

(2) $$\begin{array}{cc} \text{subject to} & \underset{k,t}{\text{∑}}I_{src_{i},\mathrm{com}p_{k},\mathrm{typ}e_{t}}+J_{src_{i}}=1\;\forall i \end{array}$$

(3) $$\begin{array}{c} \left(\underset{k,t}{\text{∑}}\Delta_{T}\cdot h_{i\to k}+\Delta_{C,t}+\Delta_{T}\cdot h_{k\to \mathrm{dst}}+\Delta_{D,t}\right)\cdot \\ I_{src_{i},\mathrm{com}p_{k},\mathrm{typ}e_{t}}+\left(\Delta_{T}\cdot h_{i\to \mathrm{dst}}\right)\cdot J_{src_{i}}+\delta \\ \leq T_{\max} \end{array}$$

where we denote *Ē_T_* as the TX/RX radio energy cost without compression and *E_T_*_,_*_t_* as the TX/RX radio energy cost after compression with type *t*. *E_C,t_* is the computation energy cost for compression with type *t*, and *E_D,t_* is the computation energy cost for decompression with type *t* at the data sink. *h_i_*_→_*_j_* is the hop distance from node *i* to node *j*; Δ*_T_* is the hop delay between TX and RX; Δ*_C,t_* is compression delay for type *t*, and Δ*_D,t_* is decompression delay for type *t*. Furthermore, some additionally incurred delay, *δ*, is considered to reflect the dynamics from the Carrier Sense Multiple Access (CSMA)-type MAC protocol into our analytical model. We use these parameters obtained from experimental measurements in Section 4.

The objective function (1) minimizes the overall energy consumption in radio and CPU computation. Constraint (2) ensures a mutually exclusive choice between compression and non-compression. Constraint (3) makes sure to satisfy the end-to-end latency requirement, *T_max_*, so that the selected compression-based delivery incurs the even earlier end-to-end delay for on-time packet delivery.

This formulation allows any sensor node to select any available compression algorithms with which to compress the data, so that it can result in the globally minimum energy consumption in the network.

#### 5.2.1. Optimization with a Limited Number of Compressing Nodes

The above formulation provides the globally optimal selection for minimizing the overall energy consumption, while there is a possibility that a subset of nodes should sacrifice additionally incurred CPU computational cost for compression for the overall network benefit. By limiting the number of compressing nodes in the network and allowing only parts of nodes to execute data compression, we aim to find a sub-optimal compression strategy.

To apply the constraint of limited compressing nodes, we apply the regularization technique [30]. Using a form of regularization is to minimize the weighted sum of the objectives: (1) the total energy cost (Equation (1)); and (2) the net number of selected nodes for any compression from any source. We introduce a new indicator function, *Ĩ_k_*, as follows:

$${\tilde{I}}_{k}=\{\begin{array}{ll} 1 & \text{if node}\;k\;\text{is selected for compression},\\ 0 & \text{otherwise}. \end{array}$$

(4) $$\begin{array}{cc} \text{minimize} & (\underset{k,t}{\text{∑}}{\bar{E}}_{T}\cdot h_{i\to k}+E_{C,t}+E_{T,t}\cdot h_{k\to \mathrm{dst}}+E_{D,t})\cdot \\ & I_{{\mathrm{src}}_{i},{\mathrm{comp}}_{k},{\mathrm{type}}_{t}}+\left({\bar{E}}_{T}\cdot h_{i\to \mathrm{dst}}\right)\cdot J_{{\mathrm{src}}_{i}}+\gamma \cdot \underset{k}{\text{∑}}{\tilde{I}}_{k} \end{array}$$

(5) $$\begin{array}{cc} \text{subject to} & \underset{k,t}{\text{∑}}I_{{\mathrm{src}}_{i},{\mathrm{comp}}_{k},{\mathrm{type}}_{t}}+J_{{\mathrm{src}}_{i}}=1\forall i \end{array}$$

(6) $$\begin{array}{c} (\underset{k,t}{\text{∑}}\Delta_{T}\cdot h_{i\to k}+\Delta_{C,t}+\Delta_{T}\cdot h_{k\to \mathrm{dst}}+\Delta_{D,t}).\\ I_{{\mathrm{src}}_{i},{\mathrm{comp}}_{k},{\mathrm{type}}_{t}}+\left(\Delta_{T}\cdot h_{i\to \mathrm{dst}}\right)\cdot J_{{\mathrm{src}}_{i}}+\delta \\ ⩽T_{\max} \end{array}$$

(7) $$I_{{\mathrm{src}}_{i},{\mathrm{comp}}_{k},{\mathrm{type}}_{t}}\leq {\tilde{I}}_{k}\forall i,\forall t$$

The regularized problem solves the bi-criterion optimization problems of making the total energy cost and the net number of selected compressing nodes small. This advanced formulation penalizes the number of compressing nodes by a factor of γ.

Constraint (7) ensures that if node *k* is selected for compression with any type from any source, *Ĩ_k_* forces it to be one, so that Σ*_k_ Ĩ_k_* is the number of compressing nodes.

In our implementation, a computer cluster connected to the sink node solves the above-formulated optimization problems efficiently by using AMPL/CPLEX [31]. In the next evaluation section, we measure computation time in a real-world computer system for the complexity of solving the binary integer programs.

## 6. Evaluation

**Figure 5.**
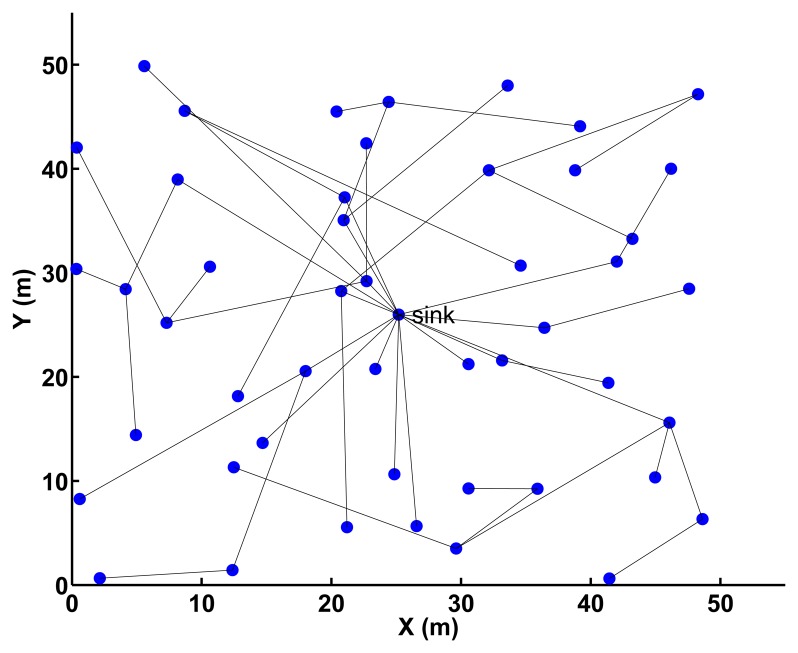
Network topology of 49 sensor nodes in a 50 × 50 m^2^ area used for experimental evaluation.

We validate our energy-optimized collection protocol with adaptive compression (CPAC) in a simulated network consisting of 49 sensor nodes in a 50 × 50 m**^2^** area (Figure 5) using the TinyOS 2.x TOSSIM simulator [32]. A state-of-the-art data collection scheme, CTP [18] is used as our underlying collection protocol.

We generate the network topology in the environment of an aisle of a building (Figure 5) using the LinkLayerModel tool in TOSSIM. We consider dynamic interference effects for more realistic simulations by applying the Closest-fit Pattern Matching (CPM) model [33]. We use lake-lagunita trace as low-level interference, meyer-light trace as mid-level interference and meyer-heavy trace as high-level interference. The meyer-light noise trace is used, unless otherwise noted.

We use the sensor datasets of the Intel Lab data [19] and SensorScope [20] to measure the compression-based algorithm performance. We use a part of the data in the normal collection mode in a learning phase when the optimized compression node and type selection (among no-compression, S-LZW and RLE-ST) are calculated based on the training data. We apply the computed optimized selection to different parts of the dataset as test data and measure the performance. The Intel Lab data is used as the default, unless otherwise noted.

In the experiments, our optimization algorithm uses the parameters of Δ*_T_* = 10 ms for computation. To deal with dynamic route variations in the network, we continuously compute the optimization of the compression decision (in Section 5.2) from 10 consecutively received packets in the learning phase and combine the compression strategy tables into a table. When multiple compression types are assigned to a node, we update with the less intensive compression algorithm to ensure that latency constraints can be satisfied with a large margin.

We compare the network performance of data collection for: (1) the unmodified CTP collection (no-opt (no optimization)); and (2) our optimized CPAC collection (opt), in terms of energy consumption, the packet delivery ratio, delay and overhead performance. When we measure energy consumption, we exclude other CPU energy other than compression and decompression. Since this equally affects both no-opt and opt, it does not change the comparison results. Further, in an experiment, we investigated if the CPU energy consumption for other computation jobs, such as reading the header and acting accordingly, is measured as three orders of magnitude less than compression algorithms and, thus, is negligible. While all of 48 data source nodes send 30 consecutive packets to a data sink node, respectively, we use mean values of the 30 packet transmissions for the statistical significance of our results.

**Figure 6.**
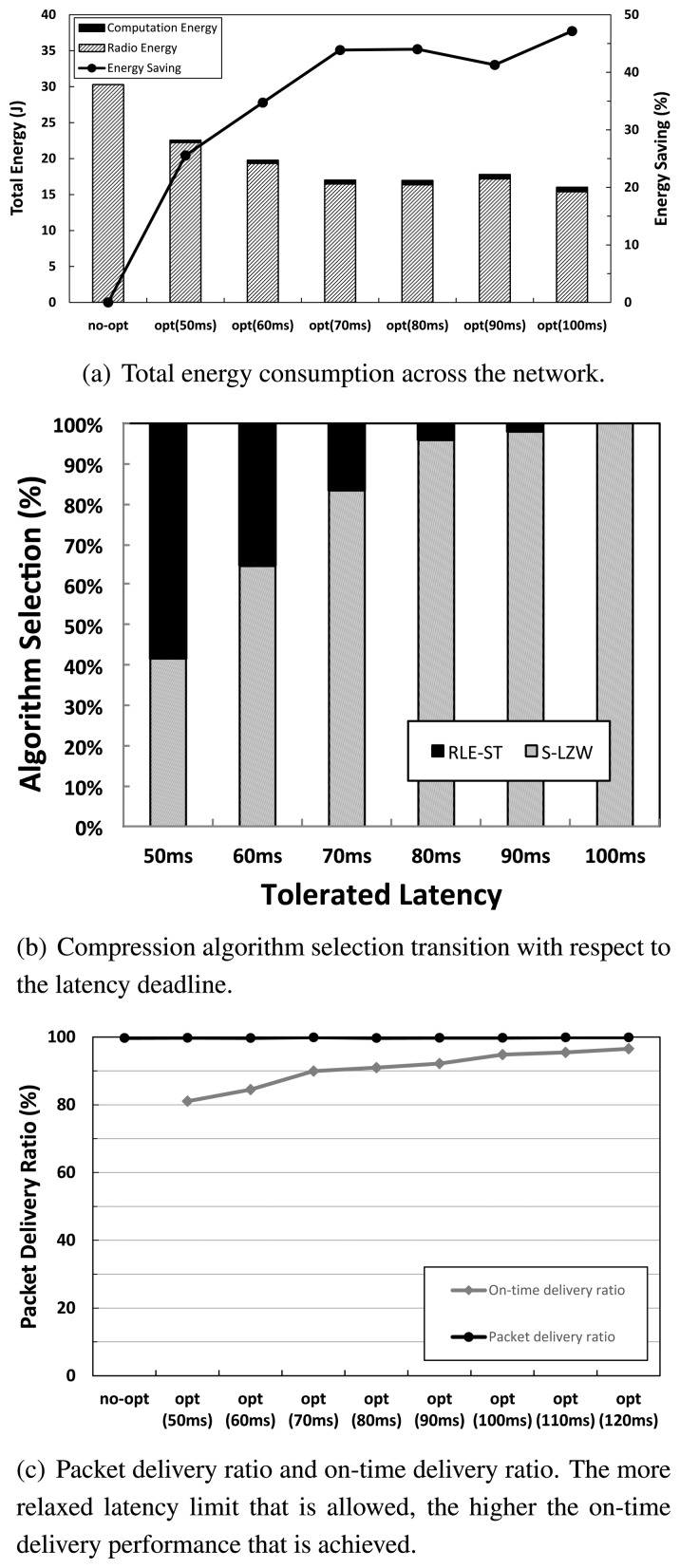
Network performance for normal collection (no-opt (no optimization)) *vs.* optimized collection (opt) with respect to latency deadline *T_max_*.

First, we evaluate the network performance of the unmodified CTP collection (no-opt) and our CPAC collection (opt), as the latency limit is varied in Figure 6. If data sources are allowed to deliver data to the sink node within a latency limit, our algorithm finds a very effective way of using selective compression, leading to an energy savings of up to 47% (for packet delivery cases with latency deadlines larger than or equal to 100 ms), as in Figure 6a. We also analyze the percentage of compression algorithm selection as the tolerated latency increases in Figure 6b. Given a longer latency deadline, the more intensive compression algorithm, S-LZW, has been selected with a higher percentage. This is due to the fact that the resulting processing delay becomes relatively small enough to meet the latency constraint, and energy savings through taking S-LZW is maximized. Regarding packet delivery, both the no-opt and opt schemes achieve above a 99% packet delivery, while our optimization scheme achieves a high on-time packet delivery ratio of up to 94.8%, as indicated in Figure 6c. As the latency limit becomes more relaxed beyond 100 ms, an on-time packet delivery ratio of 96.5% is achieved. This shows how the allowed latency significantly reduces the overall network-wide energy consumption using our proposed optimization technique, while achieving robust packet delivery performance.

**Figure 7.**
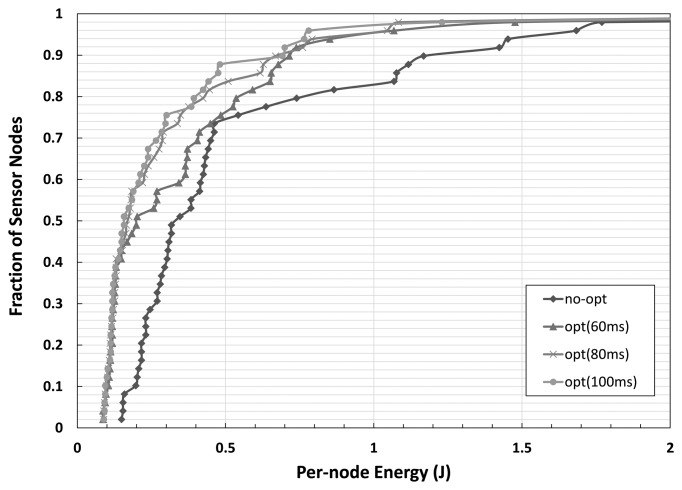
The fraction of nodes according to per-node energy consumption with respect to latency deadline *T_max_*.

We also test the per-node energy consumption of our CPAC compared to the original CTP in Figure 7. In addition to the network-wide energy savings gain, our optimization technique also improves per-node energy consumption in CTP.

**Figure 8.**
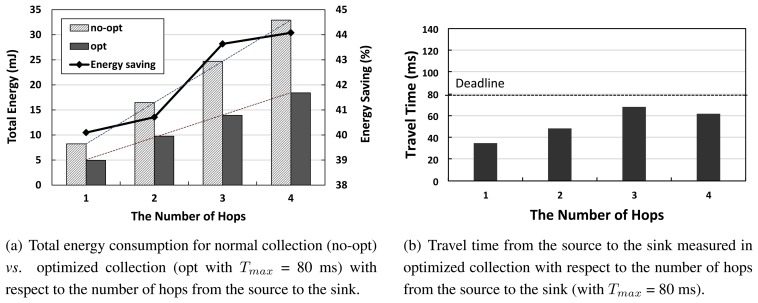
Network performance with respect to the number of hops from the source to the sink.

Next, we evaluate the network performance with respect to the number of hops from the source to the sink. This is to analyze how the network-wide energy savings gain is obtained with respect to hop distance from the source to the sink in Figure 8. As Figure 8a shows, the energy savings gain increases as the number of hops does. This is due to the following fact: without any constraint on the number of compressing nodes to use, each data source has been selected to compress its own data with a suitable algorithm, such that the given latency is considered to have been met. The amount of energy savings has been accumulated across a series of hop relays to the sink. Figure 8b shows that on-time packet delivery across a multi-hop path is achieved on average.

### 6.1. Effect of Interference Level

**Figure 9.**
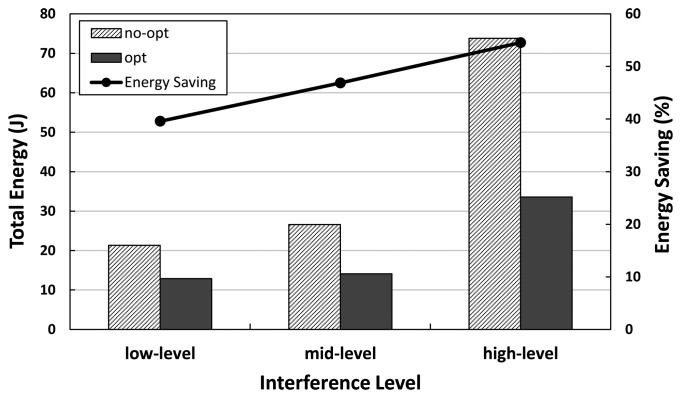
The total energy consumption of the unmodified collection vs. the optimized collection with respect to the interference level.

We investigate the effect of interference in a network environment. We take the realistic interference model in [33] from three different interference environments ((1) lake-lagunita as low-level interference; (2) meyer-light as mid-level interference; and (3) meyer-heavy as high-level interference [33]) into our evaluation. We compare total energy consumption and energy savings for no-opt *vs.* opt, with respect to the interference level in the network, as indicated in Figure 9.

As Figure 9 shows, the energy savings effect becomes more prominent as the interference level increases. This means that under a high interference environment, where a large number of retransmissions occur, our optimization technique further reduces the total energy consumption and leads to more energy savings, up to 55%.

### 6.2. Effect of Sensor Data

**Figure 10.**
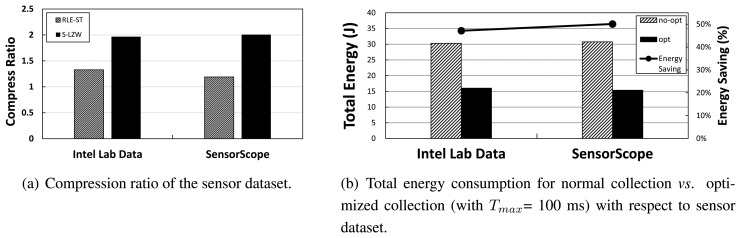
Network performance of unmodified collection vs. optimized collection with respect to sensor dataset.

Next, we explore the effect of sensor data in Figure 10. We also test total energy consumption and energy savings, depending on the sensor data: Intel Lab data *vs.* SensorScope data (see the compression ratio for a different dataset in Figure 10a). Figure 10b shows that the amount of energy savings with the usage of SensorScope data (50%) is similar to the energy savings performance based on Intel Lab data (47%). This implies that our approach is generally applicable to any real sensor data.

### 6.3. Effect of the Fraction of Compressing Nodes

**Figure 11.**
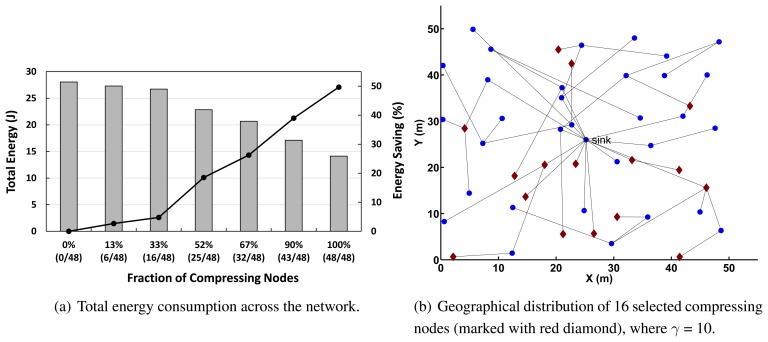
The network performance of our advanced optimization algorithm with respect to the fraction of compressing nodes.

We investigate the effect of the fraction of compressing nodes to use in our advanced optimization algorithm in Section 5.2.1. As the penalizing factor, γ, for the number of compressing nodes varies from zero to 50, we measure the total energy consumption. As the network is allowed to use a larger number of compressing nodes, our technique significantly reduces the total energy consumption, as indicated in Figure 11a (see the geographical distribution of the selected compressing nodes in Figure 11b). This means that if the network uses only a few selected nodes that execute compression, the overall network can benefit from the even limited number of resources in terms of energy efficiency.

### 6.4. Effect of Adaptive Selection

**Figure 12.**
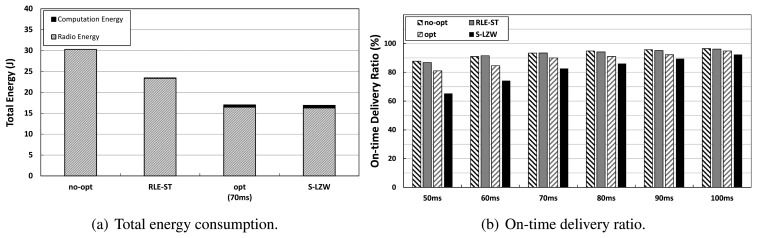
The effect of adaptive selection. Our advanced optimization algorithm, opt, is compared with only S-LZW and only RLE-ST scenarios in terms of total energy consumption and on-time delivery ratio.

We investigate how our adaptive selection algorithm performs compared to each stand-alone compression algorithm in terms of total energy consumption and the on-time delivery ratio, as in Figure 12. We conduct several experiments, such that each data source node always compresses with only S-LZW or only RLE-ST, respectively. Figure 12a shows that our opt algorithm (where *T_max_* = 70 ms) significantly reduces total energy consumption, achieving almost the same total energy as only S-LZW. As Figure 12b shows, on the other hand, the on-time delivery ratio of only S-LZW drops down to around 83%, whereas the opt scheme achieves an on-time delivery of 90%, where *T_max_* = 70 ms. Furthermore, it should be noted that the upper bound of the on-time delivery is given by the on-time delivery ratio of no-opt under the given network setup. Particularly looking at the latency of 70 ms, our opt drops only 3% compared to the upper bound in contrast to over 10% in only S-LZW. Therefore, our opt scheme is a better choice than only S-LZW in the range of tolerated latency less than the total incurred time of the packet travel time over multi-hops and the compression processing delay.

### 6.5. Overhead

We now evaluate the communication and computational complexity of our scheme. Our protocol requires dissemination of compression decision information throughout the network. Since the computed compression strategy table fits within the 114-byte payload limit in TinyOS packets, one dissemination is necessary to enable our algorithm to work in the network. We use the Drip dissemination algorithm in TinyOS 2.x. The control overhead for disseminating to 48 sensor nodes is measured as 112 packet transmissions. We believe that the incurred dissemination cost is low, since this procedure is required once in our protocol unless the network topology and the type of sensor data are substantially changed.

We also evaluate the computational complexity of our algorithm in terms of running time. We measure the running time for computing the solution of the proposed binary integer program in a computer cluster connected to data sink. In the evaluation, a Dell Precision 390 PC with a 2.66 GHz Core 2 CPU 6700 with Ubuntu 12.04 Linux is used. Table 1 shows that for the default network of 49 nodes, it takes only 212 ms, while it takes 2,152 ms in the network of 100 nodes. This demonstrates the practical feasibility of efficiently computing the proposed algorithm. In addition to this, our protocol inherently does not have any tight computation time limit: after learning from the normal data collection mode at the data sink, the network can change the mode to the optimized collection when the energy-optimal solution is computed and gets ready to be used.

## 7. Conclusion

This paper extends the problem space of data compression at local sensor nodes to a higher network-level optimization problem of joint compressing node and type selection. We have presented an energy-optimal data collection scheme by applying the selective data compression. Our approach is based on an intuition from the fundamental trade-off between latency and energy efficiency: given a longer latency limit, sensor nodes are allowed to take a more intensive compression algorithm that leads to more energy savings. Our proposed optimization algorithm provides a globally optimal solution of which compressing nodes to be selected and of which compression algorithms to be used, to reduce the overall network-wide energy consumption. Our work shows how the given tolerated delay can be turned into a network benefit of energy-efficiency through network optimization and the proposed accompanying protocol.

Our experiments indicate that our optimization technique improves the state-of-the-art collection protocol CTP, with an energy savings of up to 55% under a high-interference network, while achieving high on-time packet delivery performance. The energy savings effect becomes more prominent for a longer hop delivery by accumulating the amount of energy reduction per each hop. Furthermore, we demonstrate that only a few selected computing resources in the network can be used as substantial game changers that benefit from the overall network drastic energy savings.

Our proposed algorithm does not explicitly exploit spatial correlation over local neighboring nodes, but now only considers time correlation on the node itself. To exploit both time and spatial correlation, we would need to make local clusters of nodes in the network. By grouping several neighboring nodes into a cluster where their data are highly correlated, we can further improve network efficiency for delivering data to a data sink. This could be an interesting future work to enhance our optimization algorithm.

Currently, the proposed optimization algorithm runs on a cluster connected to the sink node and calculates a globally optimal compression decision by using the estimated delay parameters from the learning phase. Sensor nodes cannot adapt the already-made decision by considering the up-to-date remaining time and network congestion status. Furthermore, since the decision is a globally optimal solution, some of sensor nodes may be abused for the overall benefit, leading to a faster battery drain.

Considering these aspects, an interesting future direction is to design a local optimization technique by taking into account local information, such as the current battery level, the local network status and timing constraints. For this, game theoretic approaches may be complemented with our global optimization algorithm for the energy efficiency gain for both (sensor) society and the (sensor) individual.

## Figures and Tables

**Table 1.** Running time for solving our proposed optimization algorithm with respect to network size (and the number of variables).

**# of Sensor Nodes**	**# of Variables**	**Running Time (ms)**
25	1,300	24.0
36	2,664	88.0
49	4,900	212.0
64	8,320	524.0
81	13,284	1,104.1
100	20,200	2,152.1
